# Research on Decomposition of Offset in MEMS Capacitive Accelerometer

**DOI:** 10.3390/mi12081000

**Published:** 2021-08-22

**Authors:** Xianshan Dong, Yun Huang, Ping Lai, Qinwen Huang, Wei Su, Shiyuan Li, Wei Xu

**Affiliations:** 1 Science and Technology on Reliability Physics and Application of Electronic Component Laboratory, No. 5 Electronics Research Institute of the Ministry of Industry and Information Technology, Guangzhou 510610, China; laiping@ceprei.com (P.L.); huangqinwen@ceprei.com (Q.H.); suwei@ceprei.com (W.S.); lishiyuan9@163.com (S.L.); 2 Institute of Electronic Engineering China Academy of Engineering Physics, Mianyang 621900, China; xw198877@gmail.com

**Keywords:** MEMS capacitive accelerometer, offset, decomposition, parameters

## Abstract

In a MEMS capacitive accelerometer, there is an offset due to mechanical and electrical factors, and the offset would deteriorate the performance of the accelerometer. Reducing the offset from mechanism would benefit the improvement in performance. Yet, the compositions of the offset are complex and mix together, so it is difficult to decompose the offset to provide guidance for the reduction. In this work, a decomposition method of offset in a MEMS capacitive accelerometer was proposed. The compositions of the offset were first analyzed quantitatively, and methods of measuring key parameters were developed. Based on our proposed decomposition method, the experiment of offset decomposition with a closed-loop MEMS capacitive accelerometer was carried out. The results showed that the offset successfully decomposed, and the major source was from the fabricated gap mismatch in the MEMS sensor. This work provides a new way for analyzing the offset in a MEMS capacitive accelerometer, and it is helpful for purposefully taking steps to reduce the offset and improve accelerometer performance.

## 1. Introduction

With the progress of MEMS technology, the MEMS device is widely used in fields of consumer electronics, industry, and military for its small volume and low cost. The MEMS devices based on capacitance detection usually have the advantages of high accuracy and good stability, and the capacitive accelerometer is a typical representative application, which can be applied in inertial navigation and control systems [1,2]. Offset is a key issue for MEMS capacitive accelerometers, and it is the main factor blocking MEMS capacitive accelerometers from reaching higher performances [3].

Currently, the offset of MEMS capacitive accelerometers has been researched considerably, and the research has mainly involved the calibration and resource of the offset. The calibration of offset is just a mathematical operation [4,5,6]. For example, if the offset is +0.5 g and the scale factor is 0.5 V/g, then the mathematical calibration method involves subtracting 0.25 V from the output. Because the reduction isn’t from mechanism, the performance of the offset, such as drift, cannot been improved. On the other hand, there have been many studies related to the source of the offset, such as material properties [7], gap mismatch [8], residual stress [9], and sensing circuit [10], but these papers have not analyzed the offset quantitatively. Moreover, some researchers have proposed methods of reducing the offset from a mechanism, such as optimizing the adhesive process [11,12], introducing a binary-weighted micro-capacitor [13], compensating based on the resonance frequency [14], improving the control system [15], and interface circuit technology [16,17,18]. However, these methods are partial and not purposeful. At present, the method can only be arbitrarily selected to reduce the offset. For example, the method of reducing residual stress is selected to reduce the offset of a MEMS accelerometer, but its offset may mainly come from the mismatch of parasitic capacitance. Subsequently, the offset probably cannot be reduced, and the researchers think that the adopted method of reducing residual stress may not be good enough and may go in the wrong direction. Therefore, an appropriate method should be selected to reduce the offset for improving performance, which requires the decomposition of the offset quantitatively. Yet, the offset of the MEMS accelerometer originates from various factors, and it is difficult to separate the composition of the offset from each other. This leads to there being little systematic research on the decomposition of the offset. Maspero et al. analyzed the composition of the offset in a MEMS accelerometer, but this work took the MEMS sensor as a whole and could not distinguish between the device and the unavoidable parasitic term [19].

This paper proposed an offset decomposition method in a closed-loop MEMS capacitive accelerometer. Based on our developed methods of measuring key parameters of the MEMS accelerometer, the compositions of the offset were analyzed and decomposed. This decomposition method can help researchers distinguish the sources of offset quantitatively. Based on the decomposition result, the major source can be identified, and the corresponding improvement can be purposefully raised to reduce the offset. Once the offset is reduced from the mechanism level, the performance of the MEMS accelerometer can be improved. Therefore, our proposed method is of great significance for researchers to improve the performance of the MEMS capacitive accelerometer.

## 2. Decomposition of Offset

### 2.1. Composition of Offset

**Figure 1:**
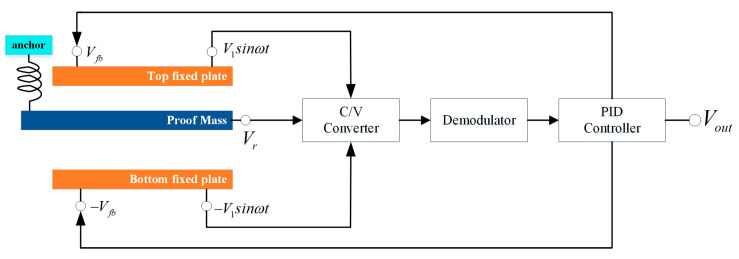
Sketch map of servo system in MEMS accelerometer.

For the closed-loop MEMS capacitive accelerometer, a sketch map of the servo system based on an electrostatic force balance is shown in Figure 1. The system contains a MEMS sensor, interface circuit, and closed-loop controller.

In the closed-loop system of the MEMS capacitance accelerometer, there is a force balance for the proof mass [20], and the offset is determined by this balance. In reference [20], the equation of force balance did not take into account the effect of residual stress. Thus, when the residual stress is considered, the equation of the force balance can be expressed as:

(1) $$F_{e}+kx+ma+F_{s}=0$$

where *k* is the stiffness of the spring, *x* is the bent value of the spring, *m* is the inertial mass of the proof mass, *a* is the input acceleration, *F_s_* is the residual stress on the inertial mass along the sensitive direction, and the equation of electrostatic force *F_e_* is as follows:

(2) $$F_{e}=F_{e1}-F_{e2}=\frac{\varepsilon_{r}\varepsilon_{0}A\times {\left(V_{d}+V_{out}-V_{r}\right)}^{2}}{2\times d^{2}}-\frac{\varepsilon_{r}\varepsilon_{0}A\times {\left(-V_{d}-V_{out}-V_{r}\right)}^{2}}{2\times d^{2}}=\frac{-2\times \varepsilon_{r}\varepsilon_{0}A\times V_{r}\times V_{out}}{d^{2}}=-\frac{V_{out}}{K_{1}}\times m$$

where *ε_r_* and *ε*_0_ are the relative and absolute dielectric constant, respectively, *A* is the overlapped area of capacitance, *V_d_* is the modulated voltage, *V_out_* is the output voltage and equal to the feedback voltage *V_fb_*, *V_r_* is the pre-load voltage, *d* is the gap between electrodes, and *K*_1_ is the scale factor and is equal to *m* × *d*^2^/(2 × *ε_r_* × *ε*_0_ × *A* × *V_r_*).

Equation (2) is substituted into Equation (1), and the formula of force balance can be transformed to:

(3) $$\frac{V_{out}}{K_{1}}=\frac{k}{m}\times x+a+\frac{F_{s}}{m}$$

On the other hand, there is a capacitance balance in the closed-loop system of the MEMS capacitance accelerometer, which means that the voltage of detecting capacitance must be equal to 0. Therefore, the equation of capacitance balance is as follows:

(4) $$(C_{T}-C_{b})\times K_{CV}=0$$

where *C_T_* and *C_b_* are the capacitances of the top electrodes and bottom electrodes, respectively, and *K_CV_* is the conversion parameter from capacitance to voltage.

The capacitances *C_T_* and *C_b_* contain the effective capacitance and parasitic capacitance. According to Equation (4), the mismatch of effective capacitance and parasitic capacitance can all result in the bending of the spring. Thus, the bent value x of the spring is determined by this capacitance balance, and the bent value of the spring can be expressed as:

(5) $$x=x_{d}+x_{p}$$

where *x_d_* is the gap mismatch due to the fabricating error and *x_p_* is caused by the parasitic capacitance and is equal to Δ*C_p_*/*K*_*x*2*C*_, where Δ*C_p_* is the mismatch of parasitic capacitance and *K*_*x*2*C*_ is the conversion parameter of the sensor from displacement to capacitance.

The offset is the accelerometer output that has no correlation with input acceleration. In additional to the MEMS sensor, the error of the circuit would also affect the offset. Thus, the effect of the circuit should also be added into the offset, and the original offset can be expressed as follows according to Equations (3) and (5).

(6) $$Offset=\frac{V_{\mathrm{out}\text{-}0g}}{K_{1}}=\frac{k}{m}\times x_{d}+\frac{k}{m}\times \frac{\Delta C_{p}}{K_{x2C}}+\delta_{e}+\frac{F_{s}}{m}$$

where *V*_*out-*0*g*_ is the output voltage without input acceleration, and *δ_e_* is the part caused by electrical components.

**Figure 2:**
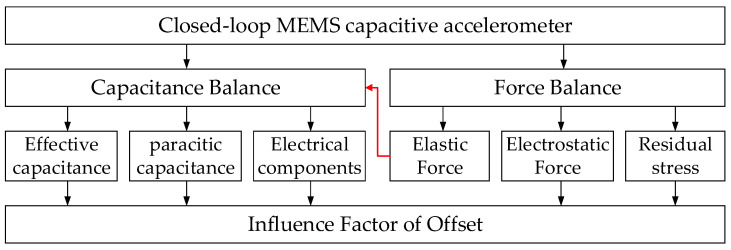
Influence factor of offset in closed-loop MEMS capacitive accelerometer.

In the closed-loop system of a MEMS capacitive accelerometer, the force balance and capacitance balance are key, and the model of the offset is deduced according to the two balances. Any factor that affects these two balances will change the offset. Figure 2 depicts the influence factor of the offset in a closed-loop MEMS capacitive accelerometer.

#### 2.1.1. Offset from Electrical Part

The offset of the MEMS capacitive accelerometer from the electrical part mainly originates from the parasitic capacitance and electrical components.

Using the method in our previous work [21], the mismatch of parasitic capacitance Δ*C_p_* can be obtained. The parameter *K*_*x*2*C*_ of the MEMS sensor can be expressed as:

(7) $$K_{x2C}=\frac{2\times \varepsilon_{0}\times \varepsilon_{r}\times A}{d_{0}^{2}}$$

Based on the parameters of *k*, *K*_*x*2*c*_, and Δ*C_p_*, the offset caused by parasitic capacitance can be separated from the whole offset.

In the open-loop system of a MEMS capacitance accelerometer, its offset is seriously influenced by electrical components, such as the front-end capacitor and the gain of an amplifier [20]. However, the closed-loop system eliminates these effects [22,23]. Meanwhile, the technology of modulation and demodulation in the front-end circuit eliminates the DC offset of the front-end amplifier [10]. However, the effect of the DC offset voltage in the PID amplifier remains. Therefore, when calculating the part of electrical components, one only needs to consider the effect of DC offset voltage of the PID amplifier, and this part can be expressed as:

(8) $$\delta_{e}=\frac{V_{os}}{K_{op}}$$

where *V_os_* is the DC offset voltage of the PID amplifier and *K_op_* is the gain of the open loop, in units of V/g. The DC offset voltage of the amplifier can be found through looking up its datasheet, and the gain of the open loop can be obtained in the open-loop system. Usually, this composition would not be large.

#### 2.1.2. Offset from Mechanical Part

The offset of the MEMS capacitive accelerometer from the mechanical part mainly originates from the gap mismatch and residual stress on the inertial mass.

To obtain the offset, which is caused by a gap mismatch, the parameters of mechanical stiffness *k* and the gap mismatch *x* should be measured. Subsequently, this offset composition of can be calculated according to *k* × *x m*.

The three-dimensional structure of a MEMS sensor is formed through the thermal bonding process, which would bring residual thermal stress, and the package would also cause mechanical stress on the sensor. This tiny stress is difficult to accurately measure, and the weight along the sensing direction is usually difficult to obtain. Therefore, it is difficult to measure this composition directly. In this paper, the value of this composition was obtained through deducting other compositions from the whole offset.

### 2.2. Parameters of MEMS Sensor

In our decomposition method of offset, the parameters of the MEMS accelerometer are the basis, including the mechanical stiffness, the gap and its mismatch, and the mismatch of parasitic capacitance. Therefore, methods of measuring these parameters were developed.

#### 2.2.1. Gap of Sensor

The actual gap between electrode plates would seriously deviate from the design value, owing to MEMS fabricating technology. Thus, the gap of the sensor should also be measured experimentally. In this paper, the value of the gap was extracted from the scale factor of the closed-loop accelerometer.

In a closed-loop system, the scale factor of the MEMS capacitive accelerometer can be expressed as [24]:

(9) $$K_{1}=\frac{m\times d_{0}^{2}}{2\times \varepsilon_{0}\varepsilon_{r}\times A\times V_{r}}$$

where *K*_1_ is a scale factor of the closed-loop accelerometer, *d*_0_ is the gap between electrode plates of the sensor, *ε_r_* and *ε*_0_ are the relative and absolute dielectric constant, respectively, *A* is the overlapped area of capacitance, and *V_r_* is the pre-load voltage. Then, the calculated formula of the gap is as follows:

(10) $$d_{0}=\sqrt{K_{1}\times 2\times \varepsilon_{0}\varepsilon_{r}\times A_{f}\times V_{r}/m}$$

#### 2.2.2. Mismatch of Parasitic Capacitance

In a MEMS capacitance accelerometer, the sensing principle is capacitance detection. Thus, the parasitic capacitance can disturb the output, and its mismatch Δ*C_p_* would result in offset. The measuring method of the mismatch of parasitic capacitance has been described in our previous work [21]. The calculating formula of mismatch parasitic capacitance Δ*C_p_* is as follows:

(11) $$\left\{ \begin{array}{l} F_{e}^{\prime }=\frac{2\times \varepsilon_{r}\times \varepsilon_{0}\times A\times x_{2}}{d_{0}^{3}}\times V_{ref}^{2}+B_{0}\\ Y=B_{1}\times X+B_{0}\\ \Delta C_{p}=\frac{\varepsilon_{r}\times \varepsilon_{0}\times A}{d_{0}-x_{2}}-\frac{\varepsilon_{r}\times \varepsilon_{0}\times A}{d_{0}+x_{2}}=\frac{2\times \varepsilon_{r}\times \varepsilon_{0}\times A\times x_{2}}{d_{0}^{3}}\cdot d_{0}=B_{1}\times d_{0} \end{array}\right.$$

where $F_{e}^{\prime }=2\times \varepsilon_{r}\times \varepsilon_{0}\times A\times V_{ref}\times V_{fb}/d_{0}^{2}=U_{out}/K_{1}\times m\times g_{L}$, $B_{0}=2\times \varepsilon_{r}\times \varepsilon_{0}\times A\times V_{d}^{2}\times x_{2}/d_{0}^{3}-k\times x-m\times a-F_{s}$.

#### 2.2.3. Mechanical Stiffness of Spring

The mechanical stiffness of spring *k* is critical for calculating the elastic force. Due to the fabricating error of MEMS technology, the actual value of mechanical stiffness would deviate largely from the design and simulation value, and so the mechanical stiffness should be measured experimentally. In this paper, a new method of measuring the mechanical stiffness was proposed and is presented below.

The mechanical stiffness was obtained through debugging the open-loop system of the MEMS accelerometer. Thus, the closed-loop accelerometer should be transformed into an open-loop system. In the open-loop system, the spring would bend with input acceleration, and there would be output voltage, while when DC voltage *V_r_* is applied to the sensor, the output would change due to the electrostatic stiffness. The difference in output voltage with different input acceleration is:

(12) $$\frac{1}{\Delta V_{out}}=\frac{-1}{m\times (a_{2}-a_{1})\times K_{x2C}\times K_{C2V}}\times k_{e}+\frac{k}{m\times (a_{2}-a_{1})\times K_{x2C}\times K_{C2V}}$$

where *a*_1_ and *a*_2_ are different input accelerations on the accelerometer, *k_e_* is the electrostatic stiffness caused by the voltage *V_r_* [25], *K*_*x*2*C*_ is the conversion parameter of the sensor from displacement to capacitance, and *K*_*C*2*V*_ is the conversion parameter from capacitance to voltage. Equation (11) can be transformed to:

(13) $$\left\{ \begin{array}{l} Y=a\times X+b\\ k=-b/a \end{array}\right.$$

where *Y* = 1/Δ*V_out_* is the dependent variable, *X* = *k_e_* is the independent variable, *a* = −1/*B*, *b* = *k*/*B*, *B* = *m* × (*a*_2_ − *a*_1_) × *K*_*x*2*C*_ × *K*_*C*2*V*_ that is a fixed value, and *k* is the mechanical stiffness.

Equation (12) shows that there is a strong linear relationship between 1/Δ*V_out_* and *k_e_*. Through linear fitting, the slope *a* and intercept b can be obtained. Then, the mechanical stiffness *k* can be obtained and is equal to −*b*/*a*.

#### 2.2.4. Mismatch of Gap

As a result of the fabricating error of MEMS technology, the gaps of the top and bottom electrode plates would be different. Consequently, the mismatch of the gap would result in an offset. The nanoscale and discreteness make the measurement of the gap mismatch difficult. Thus, a new method of measuring the gap mismatch was developed, and it is described as follows.

This method is based on the balance of electrostatic forces between top and bottom electrode plates. First, the closed-loop accelerometer is transformed into an open-loop system, and DC voltages are applied to the top and bottom electrodes. Then, adjusting the voltage amplitudes makes the output equal to the offset voltage. Under this condition, the electrostatic forces between the top and bottom electrode plates are equal. Thus, there is a balance of electrostatic forces:

(14) $$\frac{\varepsilon_{r}\varepsilon_{0}A\times V_{T}^{2}}{2\times d_{T}^{2}}=\frac{\varepsilon_{r}\varepsilon_{0}A\times V_{B}^{2}}{2\times d_{B}^{2}}$$

where *V_T_* and *V_B_* are the DC voltages applied to the top and bottom electrodes, respectively, and *d_T_* and *d_B_* are the gaps between the top and bottom electrodes, respectively. According to Equation (13), the proportion α of gaps between top and bottom electrodes and the gap mismatch *x* can be obtained, and the formulas are as follows:

(15) $$\left\{ \begin{array}{l} \alpha =d_{T}/d_{B}=V_{T}/V_{B}\\ x=(1-\alpha )\times d_{0}÷2 \end{array}\right.$$

### 2.3. Procedure of Decomposition Method

**Figure 3:**
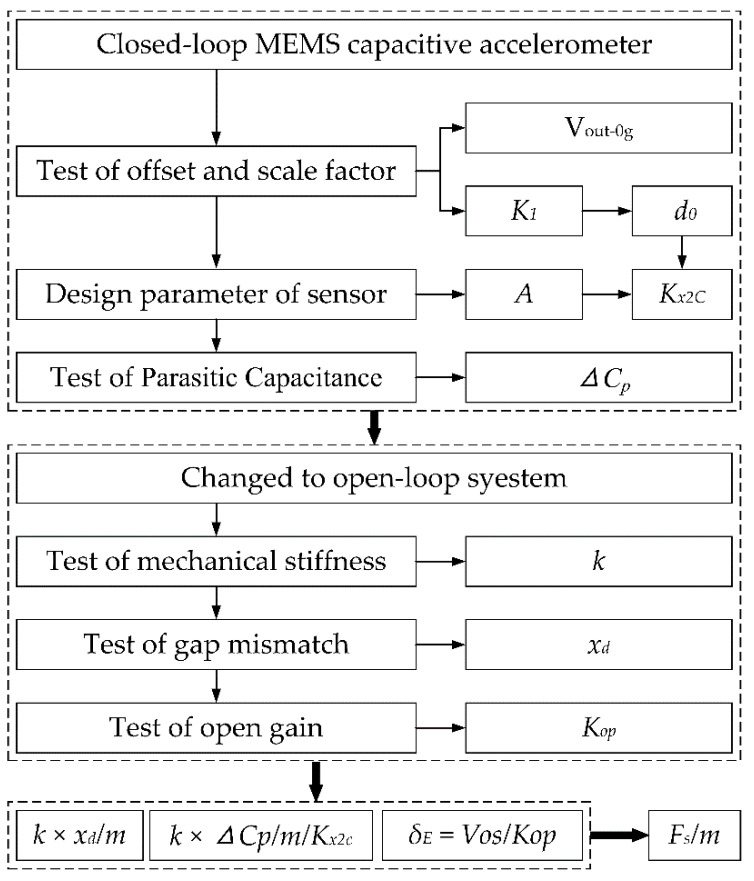
Procedure of decomposition method.

The step-by-step procedure of our decomposition of offset is described as follows, and the flowchart is sketched in Figure 3.

(1) The closed-loop MEMS capacitive accelerometer is selected as an experimental sample.

(2) In a gravity field, the offset and scale factor of the closed-loop system are tested. Based on the scale factor *K*_1_, the gap *d*_0_ can be obtained according to Equation (10).

(3) According to the design structure, the inertial mass *m* and overlapped area *A* are extracted, and some parameters can be obtained.

(4) In the closed-loop system, the test of parasitic capacitance is carried out according to the method described in our previous work [21], and the mismatch Δ*C_p_* can be obtained.

(5) The closed loop is broken at the node of the PID, and the closed-loop system is changed into an open-loop system.

(6) In the open-loop system, the test of mechanical stiffness is carried out. The DC voltages of the top and bottom fixed plates are connected to GND, and the DC voltage of the proof mass *V_r_* is disconnected from the reference fixed voltage and connected to the output of the adjustable power supply. Then, the voltage *V_r_* is changed, and the outputs of the accelerometer are recorded under the input axis conditions of vertically upward and downward. Lastly, the mechanical stiffness can be obtained according to Equation (12).

(7) In the open-loop system, the test of gap mismatch is carried out. The DC voltage of proof mass *V_r_* is connected to GND, and the DC voltages of the top and bottom fixed plates are connected to two different outputs of the adjustable power supply. The DC voltage of the top fixed plate *V_T_* is set as a fixed voltage, and the DC voltage of the bottom fixed plate *V_B_* is adjusted to make the output equal to the offset voltage. Therefore, the gap mismatch x can be obtained according to Equation (14).

(8) In the open-loop system, the test of the open gain is carried out in a gravity field. The DC voltage of the proof mass and fixed plates are all connected to GND, and the outputs are recorded under the input axis conditions of vertically upward and downward. The parameter *K_op_* can be obtained according to the outputs.

(9) Based on the measured data, the compositions can be directly obtained except for *F_s_/m*. The composition of residual stress is calculated through deducting other compositions from the whole offset.

## 3. Measurement Results and Discussion

### 3.1. MEMS Capacitive Accelerometer

**Figure 4:**
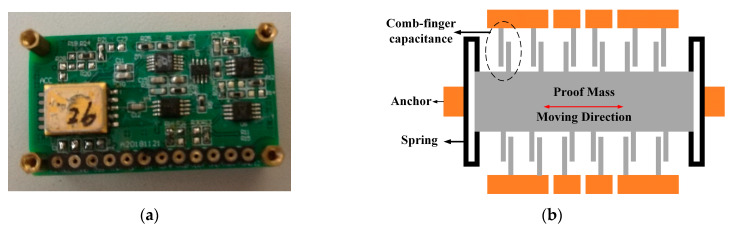
The sample (**a**) and structural diagram of sensor (**b**).

The experimental sample is our self-built MEMS capacitive accelerometer. The structure of the sensor is a comb-finger, and the inertial mass is movable in-plane. The sensor is sealed in a ceramic shell under atmospheric pressure, and the detecting circuit is constructed by discrete devices on the PCB. The sample and structural diagram of the sensor are shown in Figure 4. The servo system of this MEMS accelerometer is a closed loop based on electrostatic feedback, and a square wave of 100 kHz and 5 V is adopted in the circuit of capacitance measurement.

**Figure 5:**
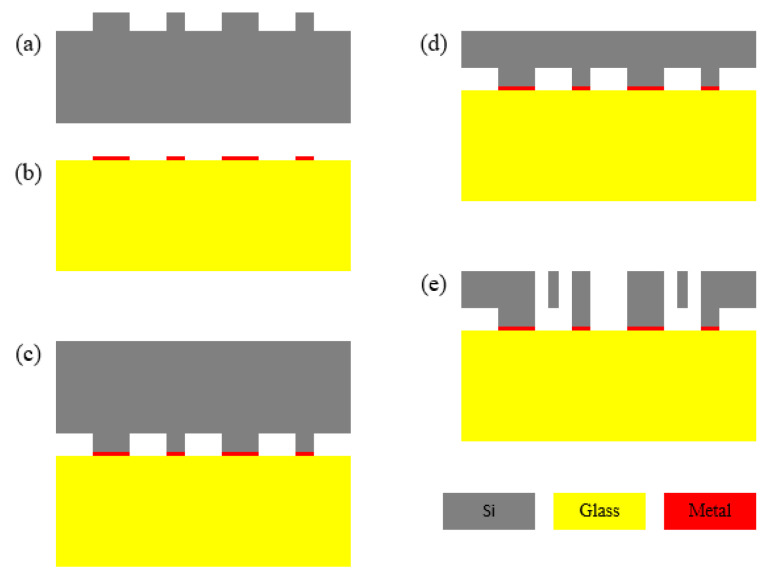
Fabrication steps of MEMS sensor (**a**) RIE on silicon; (**b**) metal patterning on glass; (**c**) anodic bonding; (**d**) silicon wafer thinning; (**e**) deep RIE on silicon.

The sensor of the MEMS capacitive accelerometer is fabricated with a bulk silicon process, and the process of SOG (silicon on glass) is adopted. The fabrication steps of the sensor are shown in Figure 5, including: (a) RIE on single-crystal silicon; (b) metal patterning on Pyrex 7740 glass; (c) anodic bonding between silicon and glass; (d) silicon wafer thinning; (e) deep RIE on silicon to release structure.

**Figure 6:**
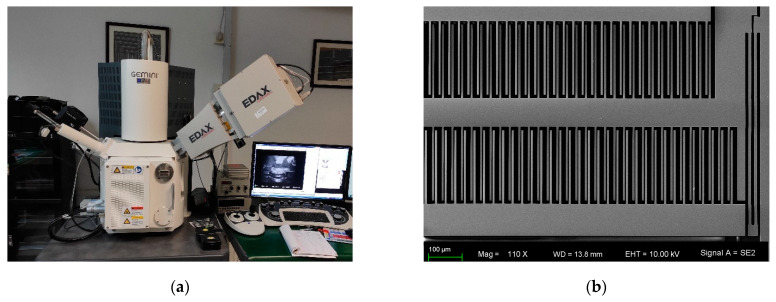
The SEM (**a**) and image of MEMS sensor (**b**).

The fabricated sensor is observed using a scanning electron microscope, and the SEM image is shown in Figure 6.

### 3.2. Measurement Results

First, the scale factor and offset of this closed-loop accelerometer were measured in a gravity field, and the data are recorded in Table 1. According to the measured data, the offset of this accelerometer was −200.4 mg, and the scale factor *K*_1_ was 578.955 mV/g.

Based on the value of the scale factor and Equation (10), the average gap of this sensor *d*_0_ could be calculated and was equal to 3.455 µm. According to the data and Equation (7), the parameter *K*_*x*2*c*_ could be calculated and was equal to 5.281 × 10^−6^ F/m.

**Figure 7:**
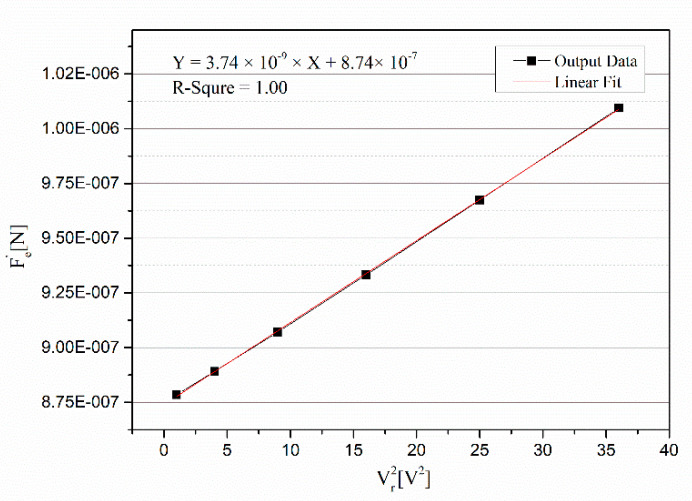
Curve of measuring the mismatch of parasitic capacitance.

In the closed-loop system, the parasitic capacitance was measured. Based on our method proposed in our previous work [21], the mismatch of parasitic capacitance was measured, and the measured data are recorded in Table 2. It should be pointed out that the controlled referenced voltage was accurate to millivolts. The curve between $V_{ref}^{2}$ and $F_{e}^{\prime }$ is shown in Figure 7, and a linear fitting of the curve was made. The linear fitting equation was *Y =* 3.74 × 10^−9^ × *X* + 8.74 × 10^−7^. According the slope of linear fitting, the mismatch of parasitic capacitance Δ*C_p_* could be obtained and was equal to 12.9 fF.

After completing the test in a closed-loop system, the closed loop was broken at the node of the PID and changed into an open loop. Then, the measurements of mechanical stiffness and gap mismatch were carried out in an open-loop system.

The measurement of mechanical stiffness was carried out based on our abovementioned method. When the DC voltage *V_r_* was applied on the middle electrode plate, the fixture with the accelerometer was overturned to make the direction of the input axis vertically upward and downward, and the outputs were recorded. Then, the amplitude of *V_r_* was changed, and the above steps were repeated. Table 3 contains the tested data where *V*_*out*1_ and *V*_*out*2_ are the output voltages of the open-loop system under the input axis conditions of upward and downward, respectively.

**Figure 8:**
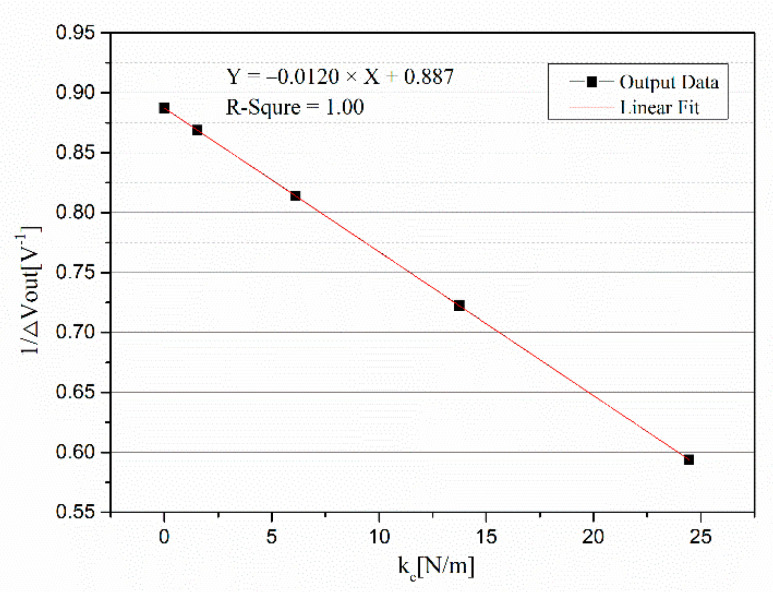
Curve of measuring the mechanical stiffness.

A curve with 1/Δ*V_out_* as the Y-axis and *k_e_* as the X-axis was made and is shown in Figure 8. The linear fitting equation was *Y* = −0.0120 × *X* + 0.887, and the *R*^2^ of the fitting was 1.00, which shows a highly linear correlation between 1/Δ*V_out_* and *k_e_*. According to Equation (12), the mechanical stiffness *k* of the spring in this accelerometer was equal to 73.92 N/m. It should be noted that the linearity would decrease when the voltage *V_r_* is larger. This is because the large displacement *x* caused by the large voltage *V_r_* would produce a nonlinear effect. On the other hand, several other samples were also tested. The linearities were all high, but they were different.

Then, the gap mismatch was also measured in an open-loop system. In this measurement, the input acceleration should be as small as possible, ensuring that the spring is in a free state, which can improve the accuracy of measurement. Thus, we adjusted the position of the accelerometer so that the value of the output was −116.0 mV. At that position, the voltage *V_B_*, with a value of 1 V, was applied on the bottom electrode, and the voltage *V_T_* was then applied on the top electrode and adjusted. The tested data are shown in Table 4.

According to Equation (14), the proportion α of gaps between the top and bottom electrode plates was equal to *V_T_/V_B_* = 1.008, and the gap mismatch was *x* = (1 − *α*) × *d*_0_/2 = −0.01382 µm. When the loop of the servo system was closed, the spring would bend due to the fabricated gap mismatch, and the bending value was equal to *x*. Thus, the offset caused by the fabricated gap mismatch was equal to *k* × *x/m* = −222.8 mg.

Lastly, the offset caused by the DC offset voltage in the PID amplifier was analyzed. The type of PID amplifier was OP4177 in this accelerometer, and its DC offset voltage was smaller than 60 µV. The measured gain of the open loop was 103.823 mV/g, and so the offset caused by this voltage was smaller than 0.6 mg according to Equation (8). Therefore, this composition was small enough to be ignored.

### 3.3. Discussion

Our proposed decomposition method of offset takes advantage of the characteristic that the MEMS sensor has a movable structure, and electrical methods were exploited to nondestructively measure the parameters of the accelerometer. Our proposed methods of measuring the accelerometer key parameters are based on our decomposition method of the offset, and it can also be extended to other applications, for example, the evaluation of MEMS fabricating technology and the design of a control system.

The real values of the accelerometer key parameters are difficult to obtain exactly. For example, the structure of the sensor has hundreds of comb-fingers, which make the equivalent gap difficult to obtain exactly. The micro-scale also makes the mechanical stiffness difficult to obtain exactly. This fact prompted us to develop new methods. In order to verify the accuracy of the results obtained by our proposed methods, another approach was introduced, and measurements were carried out.

**Figure 9:**
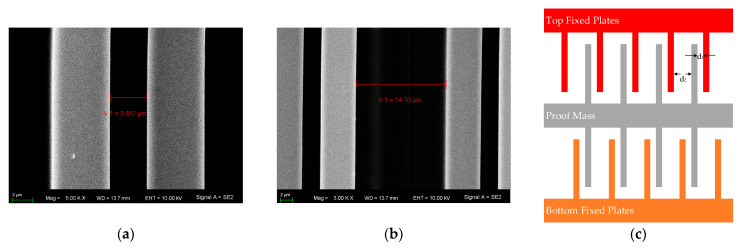
Experimental measurement of gap: (**a**) confocal laser scanning microscope; (**b**) measurement results; (**c**) capacitance model.

In this experimental MEMS sensor, the gap was designed to be 2.5 µm, and the over-etching width was about 1 µm in this SOG process. Based on our electrical measurement method, the fabricated gap of the MEMS sensor was 3.455 µm. Moreover, these data were compared with the result of dimension measurement. The SEM was used to experimentally measure the gap of the comb. The result of one comb is shown in Figure 9a,b, and *d*_1_ was 3.617 µm and *d*_2_ was 14.70 µm. According to the capacitance model shown in Figure 9c, the equivalent gap was equal to:

(16) $$d_{0}=1/\sqrt{\left(1/d_{1}^{2})+(1/d_{2}^{2}\right)}$$

Thus, the equivalent gap measured by SEM was 3.499 µm. The result of dimension measurement was close to the result of our algorithm, and the difference was 1.3%, which mainly comes from the following two aspects: (1) The sensor has several hundreds of combs, and the gap difference of different combs can reach up to 0.1 µm. (2) During the dimension measurement, an unclear boundary can lead to measurement error.

The MEMS etching process also resulted in a gap mismatch with a value of −0.01382 µm. This value seems very small, but it introduced a major offset. Similarly, the mechanical stiffness deviated from the design value by the MEMS etching process. The measured value of mechanical stiffness was 73.95 N/m, and it was much smaller than the design value of 109 N/m. Using the confocal laser scanning microscope, the width of the spring was measured, and the value was 8.8712 µm. According to the theoretical formula of mechanical stiffness for the folded beam, the calculated value was 76.21 N/m, which is close to the result of our algorithm. The difference of 3.1% mainly came from the nonuniformity of the several beams and simplified theoretical formula.

On the other hand, the error of the dimensional measurement using SEM was 0.1%, and the error of the source voltage was 0.2%. Based on these errors, the uncertainty in different items for the offset compositions can be obtained. The uncertainty in the first item $k/m\times x_{d}$ was 3.1%. The uncertainty in the second item $k/m\times \Delta C_{p}/K_{x2C}$ was 4.6%. The third item $\delta_{e}$ was small enough to be ignored. The uncertainty in the second item $F_{s}/m$ was 7.7%.

Based on our analysis and methods, the offset of the MEMS capacitive accelerometer was successfully separated from each other using our proposed decomposition method. For example, the offset caused by the mismatch of the parasitic capacitance was *k* × Δ*C_p_/K_x2c_/m* = + 39.5 mg. The separated compositions are summarized in Table 5. The composition caused by residual stress was calculated through deducting other compositions from the whole offset. As our proposed methods do not contain a method of measuring residual stress and its effect, we cannot obtain the value of this composition directly at present. In our future work, we will develop a method of directly measuring the residual stress and corresponding offset.

As can be seen from Table 5, the mechanical part with a value of −240.5 mg was much larger than that of the electrical part of +40.1 mg. Moreover, the major offset was from the fabricated gap mismatch. Therefore, the weights of different compositions revealed the primary improvement direction to reduce offset. In our next work of improving the offset performance, we will focus on reducing the gap mismatch from the etching process and structural design.

## 4. Conclusions

This paper describes a decomposition method of offset in a closed-loop MEMS capacitive accelerometer. The model of the offset was established, and the effect of the mechanics and electricity were analyzed. Methods of measuring the mechanical stiffness and gap mismatch in the MEMS capacitive accelerometer were first introduced and validated. The experiment of our silicon-based comb-finger MEMS accelerometer showed that the offset was successfully decomposed, and its source was mainly from the fabricated gap mismatch. Our future work may focus on reducing the gap mismatch and developing a method of measuring the residual stress based on the decomposition result. This work is helpful for designers to distinguish the source of offset in MEMS capacitive accelerometers and purposefully take steps to reducing the offset for improving performance.

## Figures and Tables

**Table 1:** Measured data of scale factor and offset.

*V_out_*(+1g) [mV]	*V_out_*(−1g) [mV]	SF [mV/g]	Offset [mV]	Offset [mg]
−694.96	462.95	578.955	−116.005	−200.4

**Table 2:** Data of measuring mismatch of parasitic capacitance.

$V_{ref}[V]$	$V_{r}^{2}[V^{2}]$	$F_{e}^{\prime }[N]$
1.000	1.000	8.79 × 10^−7^
2.000	4.000	8.89 × 10^−7^
3.000	9.000	9.07 × 10^−7^
4.000	16.000	9.33 × 10^−7^
5.000	25.000	9.67 × 10^−7^
6.000	36.000	1.009 × 10^−6^

**Table 3:** Tested data of measuring mechanical stiffness.

*V_r_* [V]	*k_e_* [V]	*V*_*out*1_ [V]	*V*_*out*2_ [V]	Δ*V_out_* [V]
0.000	0.0000	−0.6627	0.4644	1.1271
1.000	1.529	−0.6769	0.4740	1.1509
2.000	6.114	−0.7240	0.5045	1.2285
3.000	13.757	−0.8182	0.5661	1.3843
4.000	24.458	−1.0006	0.6836	1.6842

**Table 4:** Tested data of measuring gap mismatch.

*V_B_* [V]	*V_T_* [V]	*V_out_* [mV]
0.000	0.000	−116.0
1.000	0.000	−282.2
1.000	1.000	−118.1
1.000	1.008	−116.0

**Table 5:** Decomposition results of offset.

Composition of Offset		Value
mechanical part	gap mismatch	−222.8 mg ± 6.9 mg
	residual stress	−17.7 mg ± 1.4 mg
electrical part	parasitic capacitance	+39.5 mg ± 1.8 mg
	electrical components	+0.6 mg
total		−200.4 mg

## Data Availability

Not applicable.
